# Prevalence, transitions and factors predicting transition between frailty states among rural community-dwelling older adults in Malaysia

**DOI:** 10.1371/journal.pone.0206445

**Published:** 2018-11-05

**Authors:** Nur Sakinah Ahmad, Noran Naqiah Hairi, Mas Ayu Said, Shahrul Bahyah Kamaruzzaman, Wan Yuen Choo, Farizah Hairi, Sajaratulnisah Othman, Norliana Ismail, Devi Peramalah, Shathanapriya Kandiben, Zainudin Mohd Ali, Sharifah Nor Ahmad, Inayah Abdul Razak, Awang Bulgiba

**Affiliations:** 1 Centre for Epidemiology and Evidence Based Practice, Department of Social and Preventive Medicine, University of Malaya, Kuala Lumpur, Malaysia; 2 Division of Geriatrics, Department of Medicine, Faculty of Medicine, University of Malaya, Kuala Lumpur, Malaysia; 3 Department of Primary Care Medicine, Faculty of Medicine, University of Malaya, Kuala Lumpur, Malaysia; 4 Tobacco Control Unit, Ministry of Health Malaysia, Putrajaya, Malaysia; 5 Negeri Sembilan State Health Department (JKNNS), Negeri Sembilan, Malaysia; University of Brescia, ITALY

## Abstract

**Objectives:**

This study aims to describe the prevalence and transitions of frailty among rural-community dwelling older adults in Malaysia and to analyse factors associated with different states of frailty transition. Frailty was conceptualized using modified Fried phenotype from the Cardiovascular Health Study.

**Design:**

This is a prospective longitudinal study with 12-months follow up among older adults in Malaysia.

**Setting:**

Kuala Pilah, a district in Negeri Sembilan, which is one of the fourteen states in Malaysia.

**Participants:**

2,324 community-dwelling older Malaysians aged 60 years and older.

**Results:**

The overall prevalence of frailty in this study was 9.4% (95% CI 7.8–11.2). The prevalence increased at least three-fold with every 10 years of age. This increase was seen higher in women compared to men. Being frail was significantly associated with older age, women, and respondents with a higher number of chronic diseases, poor cognitive function and low socioeconomic status (*p*<0.05). During the 12-months follow-up, our study showed that the transition towards greater frailty states were more likely (22.9%) than transition toward lesser frailty states (19.9%) while majority (57.2%) remained unchanged. Multivariate logistic regression analysis showed that presence of low physical activity increased the likelihood of worsening transition towards greater frailty states by three times (OR 2.9, 95% CI 2.2–3.7) and lowered the likelihood of transition towards lesser frailty states (OR 0.3, 95% CI 0.2–0.4).

**Conclusion:**

Frailty is reported among one in every eleven older adults in this study. The prevalence increased across age groups and was higher among women than men. Frailty possesses a dynamic status due to its potential reversibility. This reversibility makes it a cornerstone to delay frailty progression. Our study noted that physical activity conferred the greatest benefit as a modifiable factor in frailty prevention.

## Introduction

An ageing population is a universal phenomenon experienced by all countries in the world [1]. Occurring at different paces in different settings, this demographic transition increases the number of older adults in most countries [2]. This transition has profound implications as an ageing population is associated with functional decline which leads to higher dependency. Following this scenario, common geriatric syndromes such as frailty have been given special attention [3, 4].

Frailty in the simplest definition is increasing vulnerability to adverse health outcomes. Theoretically, it is a medical syndrome with a state of increased vulnerability to stressors that result from decline in physiological reserve and function [5, 6]. The presence of frailty among older adults indicates multisystem dysregulation resulting in decreased adaptability and loss of homeostatic mechanisms [7, 8]. These processes comprise the capacity to withstand environmental stresses and thus expose these older adults to an increased risk of adverse outcomes [9–11]. Given that the consequences of cumulative decline involve multiple physiological systems, frailty has been recognised as the most problematic expression of an ageing population [3].

To date, there is no established gold standard in assessing frailty [12]. Frailty was initially measured using a single-dimensional construct where the measurement was oriented mainly to the physical domain of frailty in the original Cardiovascular Health Study (CHS) by Fried et al. [9]. Ever since it was introduced, the frailty scale developed by Fried et al. has been extensively tested for its validity [13]. Frailty has also been measured as a multidimensional construct using the Frailty Index (FI) as proposed by Rockwood et al. in the Canadian Study of Health and Aging (CSHA) [14]. Nevertheless, despite varied approaches and tools used in defining frailty, both concepts were found to be associated with adverse health outcomes, such as disability, falls and death among older adults [15].

Globally, studies related to frailty have increased tremendously. Worldwide prevalence of frailty varies from 5.8% to 35.0% [16] with overall weighted prevalence of 10.7% (95% CI 10.5–10.9) [17]. Despite the growing attention, evidence on frailty in Malaysia is scarce and limited. One study reported a 5.7% prevalence of frailty in Malaysia using the multidimensional approach [18], while another study using Fried’s phenotype reported an 8.9% prevalence [19].

Many studies consider frailty as a continuum process that involves transition towards worsening or improving states [20]. This likelihood of transitioning between frailty states provides an opportunity for prevention and remediation of frailty. Although transition between frailty states has been reported from previous studies [21–23], little is known about the likelihood of transition towards different frailty states [21]. In addition, research on frailty transition among Malaysian community-dwelling older adults is relatively unexplored. Due to the potentially reversible concept of frailty, this justifies the focus and attention to learn more about frailty and its transition states among the older population. Therefore, this study aims to: 1) describe the prevalence of physical frailty and its transition states; 2) determine factors associated with different states of frailty transitions.

## Materials and methods

### Study design

This study is part of a longitudinal cohort study among older adults in Kuala Pilah, Negeri Sembilan established since 2013.The study population includes older adults aged 60 years and older, residing in Kuala Pilah. Baseline data collection was conducted from November 2013 to February 2014 and a 12-month follow up was carried out from December 2014 to January 2015.

### Setting

Kuala Pilah is one of the seven districts in a state called Negeri Sembilan which reported the highest prevalence of peripheral muscle wasting (40.7%) nationwide among older adults aged 60 years and older [24]. Mainly rural, Kuala Pilah is situated 100 km away from the capital city of Kuala Lumpur.

### Sampling strategy

A two-stage cluster sampling method was employed in this study using a comprehensive sampling frame from the 2010 National Population Census Report prepared by the Department of Statistics, Malaysia (DoS). The DoS randomly chose 156 enumeration blocks (EB) from the existing 254 EBs in Kuala Pilah followed by another random selection of sixteen living quarters (LQs) from a total of 80 to 120 living quarters in each of these EBs using a computer-generated list.

### Participants’ inclusion and exclusion criteria

**Fig 1:**
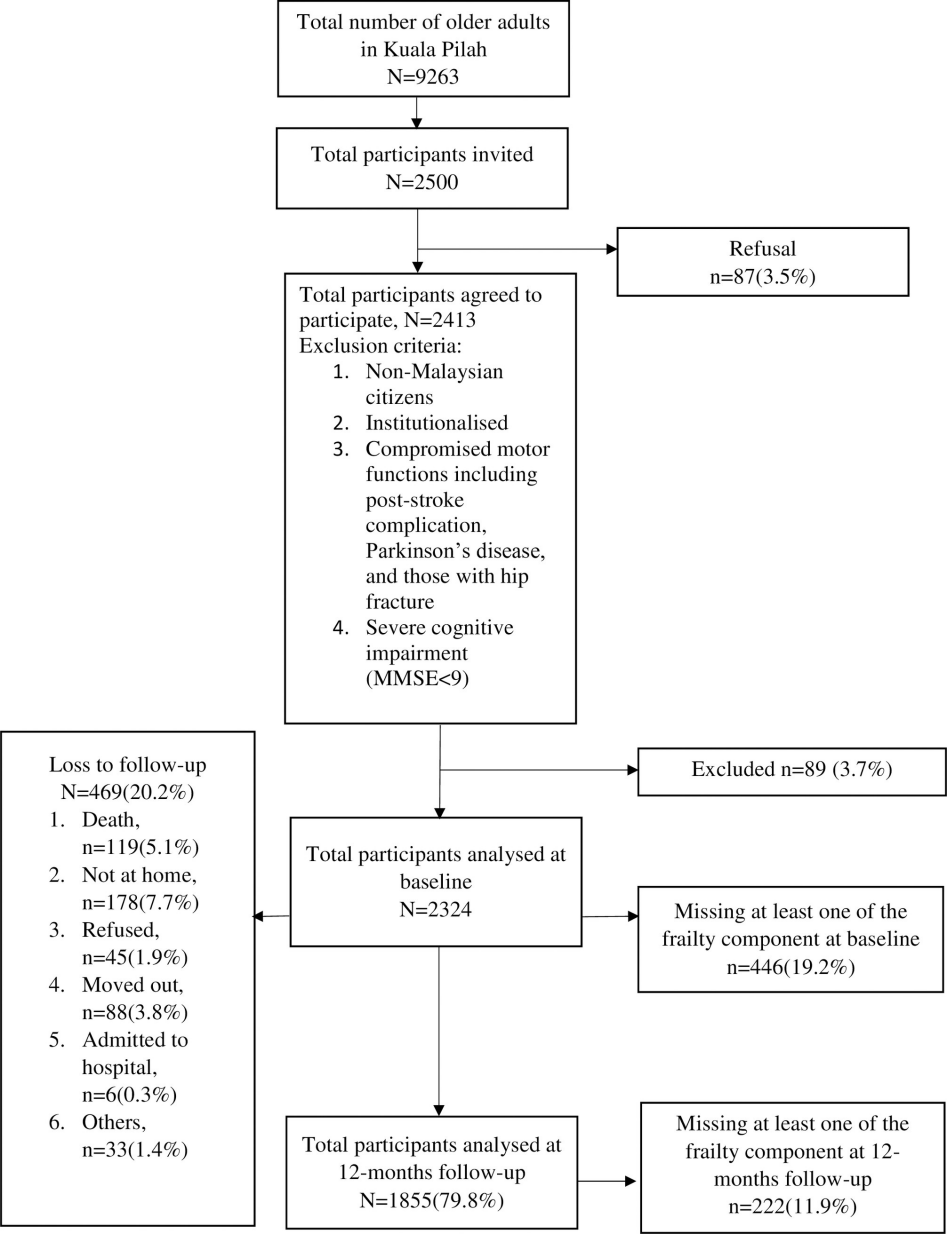
Study flow chart.

Eligible participants were those aged 60 years and older during baseline data collection and residing in Kuala Pilah district. The cut-off age of 60 years is defined based on the national guidelines of older adults in Malaysia [1]. Their age was verified using the date of birth stated in their identity cards. Participants were excluded if they were non-Malaysians, institutionalised, unable to walk independently or were having compromised motor functions. We defined compromised motor functions as having one of the following conditions: a) post-stroke complications; b) Parkinson’s diseases; c) individuals with hip fracture. We excluded individuals with these conditions as they were most likely unable to be objectively assessed for hand grip strength and walking time as part of frailty components. We included those with major cognitive impairments to determine the association between different degree of cognitive status with frailty transition states. We only excluded respondents with severe cognitive impairment (Mini-Mental State Examination score <9) [25]. Interviews and measurement of physical frailty components were conducted in the participants’ own homes by trained personnel which comprised four teams. Each team headed by a physician and three graduate research assistants. All personnel received training for geriatric assessment by the physician prior to the data collection. A total of 2,324 participants were recruited during the baseline phase while 1,855 were reassessed during the 12-month follow up phase. Fig 1 illustrates the study flow chart.

### Measures

#### Physical frailty

Physical frailty was conceptualized based on the modified Fried’s phenotype (FP) from the CHS study which consisted of five components: weight loss, exhaustion, low physical activity, weakness, and slowness [9]. Both weakness and slowness components were operationalized according to the CHS study. The remaining three components were operationalized with some adaptations: a) weight loss and low physical activity components, were based on the Concord Health and Aging in Men Project (CHAMP), a large cohort study on frailty in Australia [26–28], and; b) exhaustion component was defined according to the Study of Osteoporotic Fractures (SOF)[29]. These adaptations are due to the availability of data in our study. The details of the five components are as described below:

1. Weight loss: Defined as the respondent’s current weight which was at least 15% less than the lifetime maximum weight (taking as self-reported weight at 25 years old) [26]. Current weight was measured during the interview. Given our reference to CHAMP study for this operational definition, we did not determine whether the weight loss was intentional or unintentional.
2. Exhaustion: Self-reported exhaustion was identified by a question from the Geriatric Depression Scale (GDS) “Do you feel full of energy?”. Participants who responded “No” to this question were classified as exhausted [29].
3. Low activity: Assessed using the Physical Activity Scale for the Elderly (PASE) tool and the lowest quintile designated low physical activity [26].
4. Weakness: Measured using Jamar dynamometer with participants performing two trials on each side. The mean value of the best side was used with weakness being defined as the lowest quintile of the grip strength. The value was stratified by gender and body mass index (BMI) quartiles [9].
5. Slowness: Time to walk was measured on a 4-metre course at the usual pace. Participants were asked to walk with or without walking aids. Each participant performed the walking test two times and the best walking time was taken with the lowest quintile designating slowness. The cut off values were stratified by height for each gender [9].

Scores were assigned to each frailty component (1 = Present, 0 = Absent) and summed scores were used to categorise their frailty status. Participants were classified as frail if they had three or more of the frailty components, pre-frail if they had one or two and robust if none of the components from the frailty phenotype were present [9].

#### Frailty transition

Frailty transition was defined based on changes in frailty status between baseline state and at 12-months follow-up. Transition states were classified into three categories: 1) Improved transition towards lesser frailty states (participants who changed status from frail to pre-frail or robust and prefrail to robust), 2) Worsened transition towards greater frailty states (participants who changed status from robust to pre-frail or frail and from pre-frail to frail), 3) Unchanged state (participants with similar status at the follow-up period as the baseline status) [30].

#### Other covariates

Several risk factors associated with frailty from previous studies were measured [3, 9, 31]. They are grouped into two categories:

a) Socio-demographic factors: age, gender, ethnicity, marital status, education level, household income, living arrangement and social support.

Living arrangement was grouped into two categories, living alone or living with others (including spouse, children and close relatives). Household income was measured using monthly income and categorised into: “low” (less than RM1,000), “medium” (RM1,000-RM2,499) and “high” (RM2,500 and above) [32]. Social support status was determined using the Duke Social Support Index (DSSI) [33, 34]. Total scores were divided into quartiles with those in the first quartile designated to have low social support.

b) Health status: presence of chronic diseases (include diabetes mellitus, hypertension, hyperlipidaemia, cardiovascular disease, respiratory diseases, arthritis, stroke, cancer, depression) and cognitive status.

Information on chronic diseases was self-reported by asking if the participants had ever been told by any medical personnel that they were suffering from any of the diseases listed. Cognitive status was assessed using MiniMental State Examination questionnaire. Total scores were calculated and categorised according to the guidelines [25]. All tools used in this study have been translated to the Malay language and validated for local use.

### Analytic approach

Statistical analysis was performed using Stata 14.0 statistical software (StataCorp, College Station, Texas) with a p-value of less than 0.05 considered as statistically significant. Descriptive statistics used to characterise the participants were presented in categorical variables and reported in percentages with 95% confidence intervals where appropriate. Basic characteristics of the participants at baseline were reported and stratified according to frailty status. To determine the association between frailty and risk factors, comparisons using Pearson’s Chi-square or Fisher’s exact test was used for categorical variables. Missing data at both baseline and 12-months follow-up were analysed and found to be missing at random (MAR). To attenuate biased estimates, individuals missing at least one frailty phenotype and other covariates which had missing value >5% were addressed by the multiple imputation method via chained equations (MICE). Predicting factors associated with different states of frailty transition categories were analysed using logistic regression analysis reporting odds ratios (ORs). Analysis was performed separately for the different transition categories such as improved transition towards lesser frailty states and worsened transition towards greater frailty states. Variables with *p*<0.25 at univariate analysis were considered significant and added to multivariable models for both transition categories and variables with *p*<0.05 were included in the final models. Given the complex sampling design to ensure adequate representation from the overall study population, we accounted for this by applying weightage to the selected enumeration blocks and living quarters.

### Ethics

This study was approved by the University of Malaya Research Ethics Committee(UMREC) (Ref: UM. TNC2/RC/H&E/UMREC-131) and National Medical Research and Ethics Committee, Ministry of Health Malaysia (NMRR-13-1259-16413). All participants were thoroughly briefed about the study and their written consent was obtained prior to data collection. Non-monetary incentive in form of tokens were given to all participants during baseline and follow-up period assessment.

## Results

The response rate for this study was 96.5% at baseline. Table 1 presents the basic characteristics of the participants at baseline. Prevalence of frailty and its components are reported in Table 2. A total of 9.4% of respondents were frail (7.7% in men,10.4% in women), 57.9% were pre-frail (55.3% in men, 59.6% in women), and 32.7% were robust (37.0% in men, 30.0% in women). Women reported significantly higher prevalence of low physical activity (women 26.6%, men 20.3%) and weakness (women 28.0%, men 14.2%). The prevalence of weight loss (women 8.1%, men 9.5%), exhaustion (women 32.2%, men 30.9%) and slowness (women 20.7%, men 21.1%) showed no significant difference between men and women in our study.

**Table 1:** Baseline characteristics of the study population according to frailty status.

Characteristics	All N (%)	Frailty status			Chi-square or Fisher’s p-value
		Frail, n (%)	Pre-frail, n (%)	Robust, n (%)	
Age group					
60–69	1112 (48.2)	43(19.4)	667 (49.4)	402 (53.4)	<0.001*
70–79	913 (38.7)	98(44.1)	513(38.0)	302(40.2)	
80 and above	299 (13.1)	81 (36.5)	170(12.6)	48 (6.4)	
Gender					
Men	887 (37.9)	69(31.1)	492 (36.4)	326 (43.4)	0.002*
Women	1437 (62.1)	153(68.9)	858 (63.6)	426 (56.6)	
Ethnicity					
Malay	2231 (95.6)	213 (96.0)	1296 (96.0)	722 (96.0)	0.373
Chinese	40 (1.8)	5(2.2)	18 (1.3)	17 (2.3)	
Indian	43 (2.0)	2(0.9)	31 (2.3)	10 (1.3)	
Others	10 (0.6)	2(0.9)	5 (0.4)	3 (0.4)	
Education level					
No formal education	347 (14.5)	64 (28.8)	217 (16.1)	66 (8.8)	<0.001*
Primary school	1422 (61.5)	136 (61.3)	801 (59.5)	485 (64.8)	
Secondary school	497 (21.7)	22 (9.9)	298 (22.2)	177(23.6)	
College/University	51 (2.3)	0 (0.0)	30(2.2)	21 (2.8)	
Marital status					
Married	1454 (62.4)	97 (43.9)	835 (62.3)	522 (69.8)	<0.001*
Divorce	48 (2.2)	2 (0.9)	28 (2.1)	18(2.4)	
Widowed	761 (33.4)	116 (52.5)	451 (33.6)	194 (25.9)	
Single	47 (2.0)	6 (2.7)	27 (2.0)	14 (1.9)	
Living arrangement					
Living with others	2018 (87.5)	189 (85.5)	1168(86.7)	661 (88.7)	0.281
Living alone	296 (12.5)	32 (14.5)	180 (13.3)	84(11.3)	
Social support					
Low	606 (27.6)	74 (35.2)	324 (25.7)	208 (30.4)	0.005*
High	1549 (72.4)	136 (64.8)	939(74.3)	477 (69.6)	
Household income					
Low	1504 (65.9)	172 (79.6)	888 (66.5)	444 (59.8)	<0.001*
Medium	708 (30.8)	42(19.4)	404 (30.2)	262(35.3)	
High	82 (3.3)	2(1.0)	44(3.3)	36 (4.9)	
Cognitive status					
Normal	1279 (56.6)	49 (22.9)	699 (52.7)	531 (71.9)	<0.001*
Mild	625 (27.6)	66(30.8)	406 (30.6)	153 (20.7)	
Moderate	275 (11.4)	56 (26.2)	173 (13.0)	46(6.2)	
Moderately severe	101 (4.4)	43 (20.1)	49 (3.7)	9(1.2)	
Presence of chronic diseases					
0	700 (31.5)	36 (17.6)	397 (30.3)	267 (36.6)	<0.001*
1	512 (22.6)	44 (21.5)	308 (23.5)	160 (21.9)	
2 and more	1034 (46.9)	125 (60.9)	606 (46.2)	303 (41.5)	

*p<0.05.

Weightage has been applied to the percentages to adjust for the complex sample design.

Percentages add up to 100 vertically.

**Table 2:** Prevalence of frailty and its components at baseline, overall and stratified by gender.

Frailty status	Men, % (CI)	Women, %(CI)	Total, %(CI)
Frail	7.7 (5.8–10.3)	10.4 (8.5–12.6)	9.4 (7.8–11.2)
Pre-frail	55.3 (50.6–59.9)	59.6 (55.1–63.8)	57.9 (53.9–61.9)
Robust	37.0 (32.3–41.9)	30.0 (25.6–34.9)	32.7(28.5–37.3)
**Frailty components**			
Weight loss	9.5 (7.6–11.9)	8.1 (6.7–9.7)	8.6 (7.4–10.1)
Exhaustion	30.9 (25.5–36.9)	32.2 (26.6–38.3)	31.7 (26.6–37.2)
Low activity	20.3 (17.7–23.3)	26.6 (23.4–30.0)	24.2 (21.9–26.6) *
Weakness	14.2 (11.8–16.9)	28.0 (23.9–32.6)	22.7(19.6–26.2) *
Slowness	21.1 (18.1–24.5)	20.7 (18.0–23.6)	20.8 (18.4–23.5)

*p<0.05.

CI: 95% confidence interval.

Weightage has been applied to the percentages to adjust for the complex sample design.

**Fig 2:**
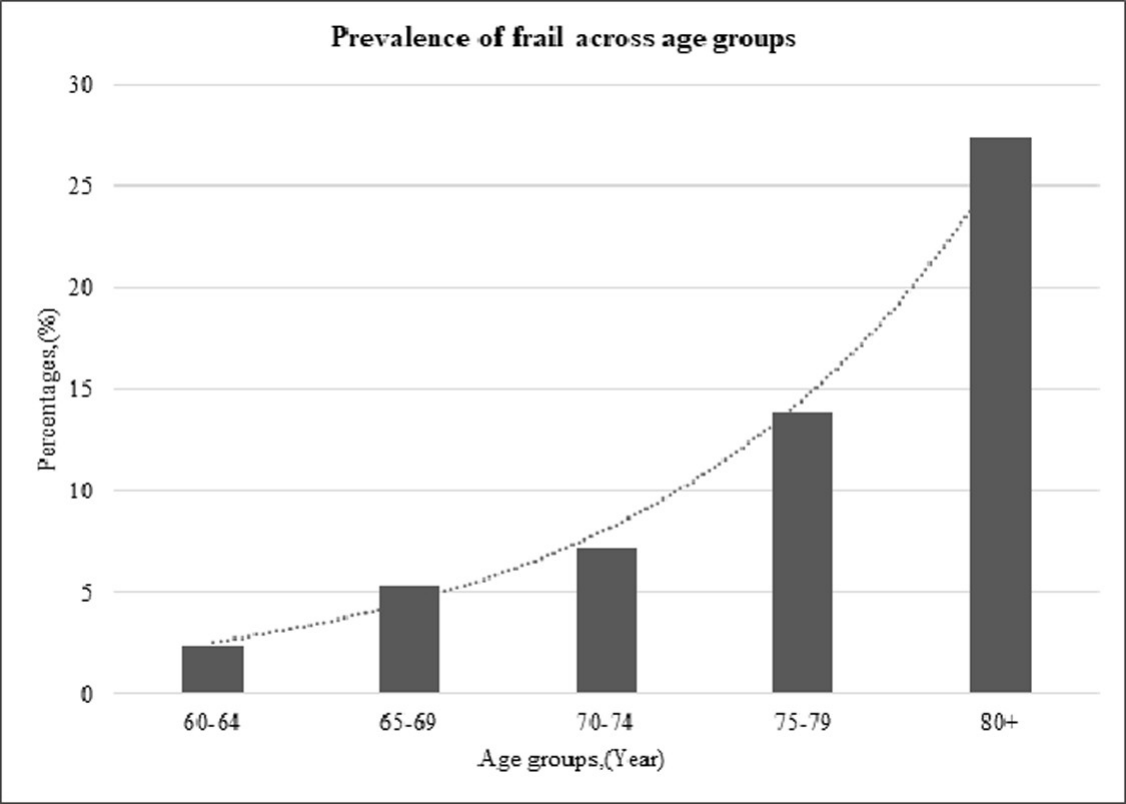
Percentage of frail category according to different age groups.

Comparing across different age groups, the prevalence of frailty increased at least three-fold for every 10 years of age (Fig 2) with the prevalence being higher in women compared to men across almost all age groups (Table 3).

**Table 3:** Frailty prevalence across age groups, overall and stratified by gender.

Age groups	Overall		Men		Women	
	n	%, (CI)	n	%, (CI)	n	%, (CI)
**60–64**	16	2.4 (1.4–3.9)	5	2.0 (0.8–5.1)	11	2.6 (1.4–4.7)
**65–69**	27	5.4 (3.7–7.8)	12	5.7 (3.4–9.4)	15	5.1 (2.9–8.8)
**70–74**	33	7.2 (5.2–9.9)	12	7.1 (3.7–13.4)	21	7.2 (4.6–11.2)
**75–79**	65	13.9 (10.3–18.4)	18	10.2 (5.9–17.0)	47	16.1 (11.4–22.2)
**80 and above**	81	27.4 (22.3–33.2)	22	19.1(12.1–28.9)	59	33.2 (25.7–41.6)

CI: 95% confidence interval.

Weightage has been applied to the percentages to adjust for the complex sample design.

Frail participants were older, more likely to be women, single or widowed, had lower education level, poor social support, low income, and lower cognitive function than those who were not frail (Table 1). They also were reported to have a higher number of chronic diseases with the majority having two and more (Table 1). All comparisons were statistically significant (*p*<0.05).

**Fig 3:**
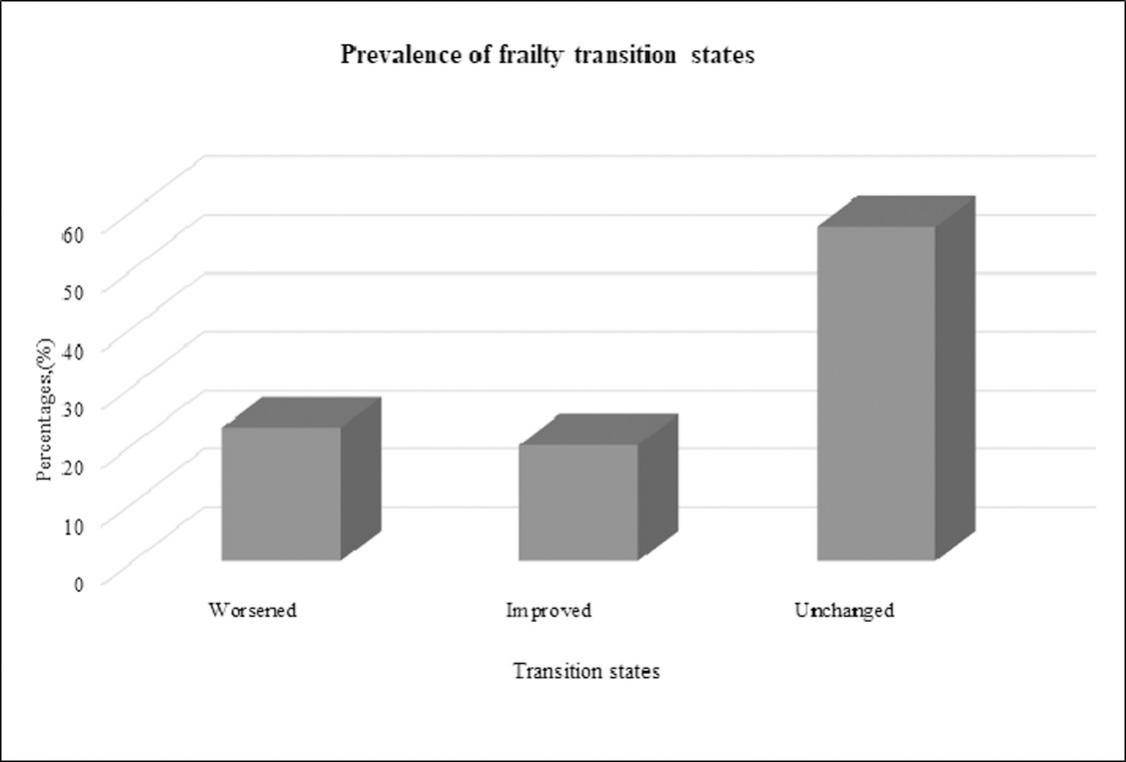
Prevalence of different states of frailty transition.

From a total of 1,855 participants with frailty measurement at both baseline and 12-months follow up, transition was observed towards greater frailty states in 22.9% of respondents, while 19.9% experienced transitions towards lesser frailty states and the majority (57.2%) remained unchanged (Fig 3). Among those who reported improved transition, 9 (6.1%) had transition across two levels, from frail to robust. Another transition across two levels was also reported from 16 participants (2.9%) who experienced a worsened transition state, from robust to frail (Table 4). Besides those who reported unchanged frailty status, the highest prevalence of transition in frailty status was from robust to prefrail (49.6%) while change from robust to frail was reported to have the lowest prevalence (2.9%).

**Table 4:** Frailty transitions states at the 12-months duration of follow up.

Baseline status	Status at 12-months follow up					
	Robust		Pre-frail		Frail	
	n	% (CI)	n	% (CI)	n	% (CI)
**Robust**	298	47.5 (42.0–53.1)	298	49.6 (44.9–54.3)	9	2.9 (1.3–6.1)
**Pre-frail**	313	28.1(24.7–31.8)	697	62.9 (59.7–66.1)	62	8.9 (7.2–11.1)
**Frail**	16	6.1 (3.2–11.4)	95	44.9 (36.1–54.1)	67	48.9 (38.8–59.3)
**Overall**	627	33.1 (29.5–37.0)	1090	57.1 (54.1–60.1)	138	9.8 (7.7–12.3)

CI: 95% confidence interval

Weightage has been applied to the sample to adjust for the complex sample design

Table 5 shows the results of regression models of the association between variables predicting different states of frailty transition. Univariate analysis on worsening transition towards greater frailty states showed that older adults having low physical activity were nearly three times as likely to worsen in frailty status (OR = 2.9,95% CI 2.2–3.7). Similarly, for improved transition towards lesser frailty states, the final multivariate regression model showed that having low physical activity lowered the likelihood of transition towards less frailty states by nearly 70.0% (OR 0.3, 95% CI 0.2–0.4).

**Table 5:** Univariate and multivariate logistic regression for variables/predictors of different states of frailty transition from baseline to 12-months.

Variables	Worsened transition to greater frailty states		Improved transition to lesser frailty states	
	Univariate	Multivariate	Univariate	Multivariate
	OR (95% CI)			
**Age**				
60–69	1.0 (Reference)	-	1.0 (Reference)	-
70–79	1.1 (0.8–1.4)	-	0.9 (0.7–1.2)	-
80 and above	1.1 (0.9–1.4)	-	0.9 (0.6–1.2)	-
**Gender**				
Men	1.0 (Reference)	-	1.0 (Reference)	-
Women	1.0 (0.8–1.3)	-	0.9 (0.7–1.1)	-
**Cognitive status**				
Normal	1.0 (Reference)	-	1.0 (Reference)	-
Impaired	1.0 (0.8–1.3)	-	0.7 (0.5–0.9) *	0.9 (0.6–1.2)
**Level of physical activity**				
Active	1.0 (Reference)	-	1.0 (Reference)	-
Low	2.9 (2.2–3.7) **	3.0 (2.3–4.1) **	0.3 (0.2–0.4) *	0.3 (0.2–0.4) **

*p<0.25.

**p<0.05.

Weightage has been applied to the sample to adjust for the complex sample design.

Multivariate model was adjusted for age and gender.

## Discussion

### Frailty prevalence and its correlates

The study found a frailty prevalence estimate of 9.4%. This is slightly higher than findings from previous local studies, which reported a range between 5.7% and 8.9% [18, 19]. The variations might be due to differences in study settings, whereby both previous studies were conducted among the urban population while the present study was among rural community-dwelling older adults. Our findings are corroborated by a study in Taiwan by Yu et al. which reported that frailty was more prevalent in the rural population compared to the urban population [35].In other studies, similar variations were also reported largely due to differences in methodology, mainly with regards the instruments used to define frailty and the types of participants [36].

Frailty was found to be correlated with age. Age-stratified frailty prevalence in this study was found to increase at least three-fold with every ten years increase in age. The prevalence increased to four-fold among older adults aged 70 years and older. This finding was supported by a systematic review and meta-analysis on the prevalence of frailty among older Japanese by Kojima et al [37].

We found that frailty was at least twice as common in women than in men across age groups, a finding that has been reported in previous studies [9, 19, 37, 38]. This phenomenon can be attributed to gender differences between men and women. First, women are at greater risks due to lower muscle strength throughout the ageing process compared to men at the same age [9]. Second, the discrepancy can be due to differences in self-reporting manner. Studies have shown that women are more likely to report a lower level of health status than men [39–41], as they are often more cautious about their health status [42]. More extensive studies across different countries in Europe also reported that frailty increased across age groups with the prevalence being higher in women than men [43].

Our study found that frailty was associated with lower socio-economic status. This finding is also similar to previous studies which described socioeconomic inequalities in frailty [9, 44, 45]. Another study on the relationship between social factors and frailty by Andrew et al. reported that social vulnerability was highest amongst the frail group [46]. Characterising risk of frailty among this vulnerable group of older adults will help in giving direction to an effective public health policy on managing frailty and reducing its burden.

### Frailty transitions and predicting factors

Previous studies have reported a higher prevalence of transition towards worsened frailty states than the transition towards lesser frailty states [21, 22]. Similarly, the present study found a higher prevalence of worsened transition towards greater frailty states compared to improved transition towards lesser frailty states. This finding was expected in view of the increasing trend of frailty prevalence across age groups [47].

Our study showed that participants with low physical activity were more likely to develop worsening transition towards greater frailty states and less likely to improve towards lesser frailty states. Previous studies had established an association between sedentary behaviour and frailty [48–50]. According to the National Health Morbidity Survey conducted nationwide, the level of physical activity gradually decreased with age and was most apparent among older adults [24]. In general, physical activity is recommended as a main non-pharmaceutical intervention among older adults [51]. This may dampen the progression of frailty by increasing the possibility of improvement among those who are frail [52]. From a total of six types of physical activities measured using PASE questionnaire (including sitting, walking, light, moderate, vigorous and strength types of exercises), our study showed that only walking had a significant association with the states of frailty transition (S1 Appendix).

While we found an association between physical activity and frailty transition states, it is uncertain which type of physical activity is the most effective for intervention[53, 54]. Previous studies have recommended different forms of physical activity, without consensus on a single type [53, 55, 56]. We thus recommend that future research should investigate the effectiveness of different types of physical activity among older adults as frailty prevention. Ultimately, the key thrust to prevent frailty is to incorporate a multicomponent exercise which addresses various domains of physical activity including strength, endurance and balance based on individual’s frailty status [57]. This requires a more structured exercise program as part of frailty management [58].

Our findings have to be interpreted in the light of several constraints. First, the study was among relatively well functioning older adults as we excluded those with severe cognitive impairment and compromised motor functions at baseline. This could have underestimated the prevalence of frailty. Second, the follow-up time to study the transition states of frailty was short relative to previous studies [21, 23, 56, 59]. Third, our operational definition used to measure frailty components in particular for weight loss, exhaustion and low physical activity were slightly different from the original CHS study. This may have affected prevalence estimates of frailty. Despite the differences, these changes were comparable to other previous large cohort studies on frailty which have also used similar definition [26–29]. In addition, using weight at 25 years of age to measure the weight loss component may have caused measurement error, but it is likely to be randomly distributed across all participants. Fourth, our study did not assess other factors associated with frailty including biological determinants such as genetic factors, hormonal changes and nutritional status which have been discussed in existing literature [60, 61].

To the best of our knowledge, this study is the first longitudinal study on physical frailty reporting frailty transition and factors associated with different states of frailty transition in Malaysia. In addition, it is also the first local study on frailty conducted among rural community-dwelling older adults. The novelty of this pioneering study adds valuable information on the burden of frailty in parallel with Malaysia’s experience on the demographic transition towards an ageing population. Other strengths include recruitment of a large sample size which is representative of the rural older population, and adoption of objective measurements of grip strength and walking speed while measuring physical frailty. Objective measurements increased accuracy of the results and reduced overestimation of these two components when compared to perceived frailty using subjective measurement [62]. Although missing data is part of our study limitation, the application of multiple imputation in handling missing data in this study reduced the bias estimates and prevents the concomitant loss of power [63].

## Supporting information

S1 Appendix Association between type of physical activities and frailty transition states. (DOCX)

S1 File Baseline data. (DTA)

S2 File 12-months follow-up data. (DTA)
