# Supplementary material for: Prevalence, transitions and factors predicting transition between frailty states among rural community-dwelling older adults in Malaysia
Source: PLoS One. 2018 Nov 5;13(11):e0206445. doi: 10.1371/journal.pone.0206445 (PMC6218037; doi:10.1371/journal.pone.0206445)
Supplement: S1 Appendix — (DOCX) [file pone.0206445.s001.docx]

**S1 Appendix. Association Between Type of Physical Activities and Frailty Transition States**

| **Variables** | **Transition states** | | | **Chi-square/Fisher’s exact test p-value** |
| --- | --- | --- | --- | --- |
|  | **Worsened, n(%)** | **Improved, n(%)** | **Unchanged, n(%)** |  |
| **Sitting activity** | | | | |
| Yes | 402(96.40) | 359(98.09) | 1028(97.26) | 0.369 |
| No | 15(3.60) | 7(1.91) | 29(2.74) |  |
| **Walking** | | | | |
| Yes | 208(49.17) | 216(58.54) | 582(54.96) | **0.033*** |
| No | 215(50.83) | 153(41.46) | 477(45.04) |  |
| **Light exercise** | | | | |
| Yes | 10(2.36) | 14(3.79) | 44(4.15) | 0.273 |
| No | 413(97.36) | 355(96.21) | 1016(95.85) |  |
| **Moderate exercise** | | | | |
| Yes | 1(0.24) | 0(0.00) | 6(0.56) | 0.326 |
| No | 422(99.76) | 369(100.00) | 1056(99.44) |  |
| **Vigorous exercise** | | | | |
| Yes | 30(7.11) | 34(9.24) | 89(8.39) | 0.531 |
| No | 392(92.89) | 334(90.76) | 972(91.61) |  |
| **Strength exercise** | | | | |
| Yes | 7(1.65) | 4(1.08) | 12(1.13) | 0.586 |
| No | 416(98.35) | 365(98.92) | 1049(98.87) |  |

**p*<0.05

Weightage has been applied to adjust for the complex sample design
